# Face processing in young adults with autism and ADHD: An event related potentials study

**DOI:** 10.3389/fpsyt.2023.1080681

**Published:** 2023-03-14

**Authors:** Ümit Aydin, Roser Cañigueral, Charlotte Tye, Gráinne McLoughlin

**Affiliations:** ^1^Social, Genetic and Developmental Psychiatry Centre, Institute of Psychiatry, Psychology and Neuroscience, King’s College London, London, United Kingdom; ^2^School of Psychology and Clinical Language Sciences, University of Reading, Reading, United Kingdom; ^3^Department of Clinical, Educational and Health Psychology, University College London, London, United Kingdom; ^4^Department of Child and Adolescent Psychiatry, Institute of Psychiatry, Psychology and Neuroscience, King’s College London, London, United Kingdom; ^5^Department of Psychology, Institute of Psychiatry, Psychology and Neuroscience, King’s College London, London, United Kingdom

**Keywords:** autism, ADHD, event-related potentials, face processing, gaze direction, emotional faces, N170, young adults

## Abstract

**Background:**

Atypicalities in perception and interpretation of faces and emotional facial expressions have been reported in both autism and attention-deficit/hyperactivity disorder (ADHD) during childhood and adulthood. Investigation of face processing during young adulthood (18 to 25 years), a transition period to full-fledged adulthood, could provide important information on the adult outcomes of autism and ADHD.

**Methods:**

In this study, we investigated event-related potentials (ERPs) related to visual face processing in autism, ADHD, and co–occurring autism and ADHD in a large sample of young adults (*N* = 566). The groups were based on the Diagnostic Interview for ADHD in Adults 2.0 (DIVA-2) and the Autism Diagnostic Observation Schedule-2 (ADOS-2). We analyzed ERPs from two passive viewing tasks previously used in childhood investigations: (1) upright and inverted faces with direct or averted gaze; (2) faces expressing different emotions.

**Results:**

Across both tasks, we consistently found lower amplitude and longer latency of N170 in participants with autism compared to those without. Longer P1 latencies and smaller P3 amplitudes in response to emotional expressions and longer P3 latencies for upright faces were also characteristic to the autistic group. Those with ADHD had longer N170 latencies, specific to the face-gaze task. Individuals with both autism and ADHD showed additional alterations in gaze modulation and a lack of the face inversion effect indexed by a delayed N170.

**Conclusion:**

Alterations in N170 for autistic young adults is largely consistent with studies on autistic adults, and some studies in autistic children. These findings suggest that there are identifiable and measurable socio-functional atypicalities in young adults with autism.

## 1. Introduction

The transition from adolescence to adulthood (i.e., young adulthood, 18 to 25 years) can highlight particular challenges for individuals with autism and attention-deficit/hyperactivity disorder (ADHD). Young adults seek to achieve independence and in doing so, they encounter an increase in social and executive demands alongside a reduction in parental scaffolding (1, 2). People with ADHD or autism have increased risk of developing a range of behavioral and cognitive problems in young adulthood, including unfavorable psychosocial outcomes, poorer academic performance and lower employment levels [see (3) for a review]. Less favorable outcomes have been reported in co-occurring autism and ADHD in comparison to a single diagnosis (4–6).

While challenges in social functioning have traditionally been more associated with autism (7), ADHD is also associated with these challenges (8). Atypicalities in social functioning continue into young adulthood in both autism and ADHD (9) and is one of the strongest predictors of poor functional outcomes for both conditions (10–13). Adequate processing of socio-emotional signals from our environment, including perception and interpretation of faces and emotional facial expressions, is crucial for appropriate adaptation to social situations and the development of interpersonal relationships (14). A thorough overview of the similarities and differences between ADHD and autism in social functioning can be found in Mikami et al. (15).

Possibly due to their evolutionary relevance, cognitive and neural processing of faces and emotional expressions occurs rapidly, reflecting prioritization in the human brain (16). Due to their fast temporal resolution, event-related potentials (ERPs) are a powerful way to examine the timing and temporal stages of face processing. A number of ERPs have been proposed to index face-sensitive activity [see (17)]. These ERPs are proposed to reflect sequential steps in visual face processing, specifically the determination of low-level features, followed by abstract structural representation of faces, and further evaluation of information, including affective valence (17, 18). In particular, the face-sensitive P1, N170, and the P300, are modulated differentially by orientations of faces (i.e., upright vs. inverted), eye gaze (direct vs. averted) and emotional facial expressions.

The N170 is one of the most studied ERPs in autism and is considered among the most promising ERPs related to the disorder (19). The N170 component is a negative waveform that peaks 130 to 200 ms after a stimulus and exhibits a larger amplitude and shorter latency to faces in comparison with other stimuli (e.g., objects) (20, 21). The amplitude of the N170 has been found to be attenuated in both autistic children (22, 23) and adults (24). Yet, a recent meta-analysis indicated that the effect size of an amplitude difference between individuals with and without autism is small and not significant across 20 studies, but it may be more sensitive to autism in adults (25). In contrast, the same study showed that a longer N170 latency is likely a promising indicator of autism across all ages (25). The relationship between the N170 and social functioning is less clear as only modest associations between the ERP and behavioral measures of social functioning have been found (26). Findings in ADHD are inconsistent, with larger N170 amplitudes found for adolescents with ADHD (27) and for adults with ADHD but only for angry faces (28). Smaller amplitudes (but only for happy faces) have been found in children with the condition (29) but again this is not consistently observed [e.g., (23)].

The earlier face-sensitive P1 component, measured at occipital sites, is believed to reflect the processing of low-level features of faces, including color and contrast and early perception of emotion (30). Studies indicate the P1 typically has a longer latency and higher amplitude for inverted compared to upright faces, possibly due to the increased difficulty of the task which thus requires more resources. Yet, this is not observed in children with ADHD who have a reduced inversion effect for latency (31). A similar atypicality in the inversion effect is seen in children with autism but for P1 amplitude (22). There may be a developmental effect for the P1 latency in autism with evidence that the P1 is delayed in autistic young adults (24), but not in children (23). The P1 also reported to be sensitive to emotion perception in adult ADHD with a reduced amplitude to negative stimuli compared to controls (28).

The relatively late central/parietal P3 is associated with more controlled contextual evaluation and is modulated by emotional expressions (32, 33). Atypicalities in P3-indexed processing in ADHD are well documented but reports on the P3 response to faces are relatively scarce with some evidence for a reduction in amplitude and latency in adults with ADHD, particularly to threat-related facial stimuli (27, 28, 34, 35). No studies to date have indicated the P3 as critical to face processing in autism (23, 36).

The aim of the present study is to investigate neural correlates of visual face processing in young adults with ADHD, autism and co-occurring autism and ADHD. Identifying objective indicators of socio-emotional functioning such as ERPs to faces and emotional expressions in young adulthood might help to understand outcomes in ADHD and autism during this critical period of development. Participants completed two passive viewing tasks: one designed to examine neural correlates to viewing upright and inverted faces with direct or averted gaze (31, 37–39); the other involved viewing faces expressing one of five emotions (disgust, fear, anger, joy, neutral) (23, 40, 41). As both tasks have been previously studied in childhood cohorts with ADHD, autism and the co-occurring conditions, this study enables an indirect comparison of face processing in childhood and young adulthood (23, 31). Due to previous findings in adults, we expected that young adults with autism will show delayed and lower amplitudes especially for the N170 component in both tasks. Diverging from most childhood findings, but similar to adult studies, we also expect lower amplitudes and longer latency for P1 in autism. Our hypothesis for the P3 component is less clear but based on previous studies, we expected this to be attenuated in ADHD, particularly for negative emotional stimuli.

## 2. Materials and methods

### 2.1. Study sample

This study was conducted with full ethical approval from King’s College London Psychiatry, Nursing and Midwifery Research Ethics Subcommittee (RESCMR-16/17-2673). All participants signed consent forms prior to participation and were from the Individual Differences in Electroencephalography in young Adults Study (IDEAS) a subsample of the Twins Early Development Study (TEDS) (42, 43).

Since the main aim of IDEAS was to understand the variation observed between ADHD and autism in young adulthood, the sample was enriched for high levels of autistic and/or ADHD traits based on childhood and adolescent measures meaning the proportion of individuals with ADHD and autism in this sample is higher than a sample selected randomly from the general population [see (9, 44) for details].

A total of 1,144 participants were invited to take part in the study from four different recruitment roads. (1) A total of 290 participants were contacted from the Social Relationships Study (SRS) phase 1, which is an established subsample of TEDS focusing on twin pairs where one or both twins met autism diagnostic criteria or displayed a subclinical autism phenotype in adolescence (45). From those 92 agreed to participate in IDEAS. (2) A total of 130 participants were invited from the Neurophysiological Study of Activity and Attention in Twins Study (NEAAT), which uses a subsample of male TEDS twins at 14 years old based on ADHD symptoms (46). From those 62 agreed to participate. (3) A total of 64 participants, who did not previously participate in SRS phase 1 or NEAAT, were invited from SRS phase 3, which was focusing on gender differences in relation to social and communication abilities and took place when the twins were aged 20 to 25. From those 48 agreed to participate. (4) A total of 660 participants were invited from the main TEDS cohort. These include people with high or low ADHD traits at ages 8–14 years old. From those 354 agreed to participate.

Overall, this community-based sample consisted of 566 (283 twin pairs) participants (271 males) with an average age of 22.44 ± 0.96 years. Ten participants were missing data for all variables included in the analyses for the present study, so the final sample used for analyses included 556 participants [please see Supplementary Material and (9, 44) for details of the sample and the exclusion criteria]. Based on the Diagnostic Interview for ADHD in Adults 2.0 (DIVA-2) and the Autism Diagnostic Observation Schedule-2 (ADOS-2), the ADHD-only group included 93 participants, the autism-only group included 31 participants, and 16 participants were in the co-occurring autism+ADHD group (with scores above the threshold in both DIVA-2 and ADOS-2), and 407 participants in the comparison group (CG) (scoring below the threshold in both DIVA-2 and ADOS-2). Table 1 indicates the demographics of the groups. Sex, but not age, was significantly different between the groups. There were more females (55.8%) than males in CG, while the percentage of females in ADHD (43.0%), autism (29.0%), and co-occurring autism+ADHD (37.5%) groups were smaller than males.

**TABLE 1:** Age and sex for each group (Comparison group, ADHD, autism, autism+ADHD) along with *p*-values for group differences.

Variable		CG	ADHD	Autism	Autism+ADHD	*P*-value
Age	Mean (SD)	22.5 (1.0)	22.2 (1.0)	22.3 (0.9)	22.3 (0.9)	0.121
Sex	Male	180 (44.2%)	53 (57.0%)	22 (71.0%)	10 (62.5%)	0.005
	Female	227 (55.8%)	40 (43.0%)	9 (29.0%)	6 (37.5%)	

### 2.2. Psychological assessments

Semi-structured in-person assessments, DIVA-2 and ADOS-2, were performed and scored by trained investigators.

Diagnostic Interview for ADHD in Adults 2.0 is used to assess ADHD symptoms (47). Nine items related to inattention and nine items related hyperactivity/impulsivity were assessed. Participants were then asked if those items cause difficulties in particular life domains. Here, we used the DSM-5 diagnostic criteria for adult ADHD, which is based on reporting five or more symptoms of inattention and/or hyperactivity/impulsivity, recall of childhood onset of symptoms, and that these cause problems in more than one life domain (48).

Autism Diagnostic Observation Schedule-2 is used to assess social and communication behaviors related to 32 autism-associated characteristics. Scores for each behavior range from 0 (minimal or no observed autistic-like behavior) to 2 or 3 (marked or definite autistic-like behavior). In this study, we used the module 4 of ADOS-2, which is designed for adolescents and adults with fluent speech (49).

Details on the administration and scoring of DIVA-2 and ADOS-2 can be found in Capp et al. (44).

### 2.3. Electroencephalography (EEG) acquisition

EEG was measured for four different tasks [expression of emotion (EoE), face-gaze, arrow flanker, cued continuous performance task] and resting state for each participant within the same session using a wireless and portable 64-channel system (Cognionics, San Diego, CA, USA; Ag/AgCl electrodes, with reference and ground electrodes placed behind the right and left ear). An online high-pass filter of 0.1 Hz was used during the acquisition and the sampling rate was 500 Hz. The order in which these tasks were delivered were counterbalanced across the whole sample (but kept constant within each twin pair). In this study the findings from two tasks related to visual face processing–here named the EoE and face-gaze tasks–were analyzed and presented.

### 2.4. ERP tasks

The EoE task, so named as it involves presentation of 10 grayscale faces of a male and a female (in young adulthood), each expressing one of five emotions (disgust, fear, anger, joy, neutral). The images were taken from the NimStim set of facial expressions (50) (see Supplementary Figure 1 for examples). On each trial a face was first presented for 700 ms and then a blue circle appeared around the face, upon which the participants were instructed to press a mouse button as soon as possible. In total the face was displayed on the screen for 1,300 ms, and all relevant ERPs were produced prior to the motor task. The screen remained dark between the trials for a duration randomly jittering between 1,200 and 1,600 ms. There were 200 trials per participant (20 trials per face/expression) The faces were standardized for size, contrast and luminosity and presented in an oval aperture that occluded sex-specific features. The stimuli were presented in a fixed sequence alternating male and female faces and avoiding close repetition of the same expression. This task has previously been used in childhood studies (23, 40).

The second task (here called the face-gaze task) involved presentation of nine color images of three female faces, three looking directly at the participant and six having an averted gaze (either looking right or left). Each of these nine faces were presented either in upright or inverted orientation on a gray background see Figure 1 in Grice et al. (BR51a,) and Figure 3 (color version is used in this study) in Farroni et al. (37) for examples (37, BR51a,). At each trial a fixation stimulus (various static cartoon images) was presented for a random duration between 800 and 1,200 ms. Then a face stimulus was presented for 500 ms followed by another 500 ms of black screen. The faces were aligned so that the eyes were at the same level as the fixation stimuli. Some of the fixation stimuli were flags and to sustain attention the participants were asked to count the number of flags presented. There were in total 360 stimuli presented in a random order in four blocks of 90. This task has been previously used in infant (37) and childhood studies (31).

### 2.5. Electroencephalography processing and ERP components

EEGLAB (52) and custom written MATLAB scripts were used for EEG pre-processing and analysis (The Mathworks, Inc., Natick, MA, USA).

Raw data was resampled to 256 Hz. Channels with a correlation of less than 0.4 with their neighbors or above 75 uV (absolute value) for more than 15% of the time were marked as bad channels. Following average referencing, Adaptive Mixture Independent Component Analysis (AMICA) was used to calculate the ICA components (53) with nsgportal plug-in on the high performance computing available on The Neuroscience Gateway (NSG)^1^ (54). Based on previous literature, ICA weights calculated using 1–30 Hz filtered data were then applied to the original EEG data filtered at 0.1–30 Hz (55). This extra step ensures a high-quality ICA decomposition while maintaining lower frequency ERP components of interest. All subsequent analysis was performed on 0.1 to 30 Hz filtered data. The Eyecatch algorithm was used for the automatic detection and removal of ICs representing ocular artifacts (56). ICA decomposition was only used to identify and remove the ocular artifacts. Continuous data was epoched −200 to 700 ms around the stimulus (appearance of the face), baseline corrected using the prestimulus interval −200 to 0 ms, and epochs exceeding an amplitude threshold (± 100 uV) were removed. Bad channels were interpolated using spherical interpolation. Only EEG data with at least 20 clean epochs for any condition were used in further analysis resulting in valid ERP data for 517 participants for the face-gaze and 515 participants for the EoE task. There were no statistically significant differences between groups in terms of number of trials (see Supplementary Table 1 for the average number of epochs retained for each group and condition).

In this study we used peak amplitudes of ERP components to allow a more direct comparison with relevant childhood studies (which have also used peak amplitudes) and to investigate latencies in addition to amplitudes (23, 31). The parameters for peak amplitudes and latencies of P1, N170, and P3 ERP components were based on previous studies (23, 31). To determine exact time intervals, the peak for each component was calculated from the grand averaged EEG signals over all subjects regardless of their group. Intervals around these peaks were selected and individual participant signals were plotted to ensure that the selected interval covered individual peaks. Following this procedure, the P1 was determined as the maximum at O1 and O2 electrodes within time interval 94–195 ms for face-gaze and 105–207 ms for EoE, N170 is the minimum at P7 and P8 electrodes within time interval 125–273 ms for face-gaze and 129–277 ms for EoE, and P3 is the maximum at Pz electrode within time interval 191–339 ms for face-gaze and 273–422 ms for EoE. Please note that due to the 256 Hz sampling rate, samples occurred every 3.9 ms, restricting the interval endpoints, which precluded using exact multiples of 5 or 10 ms intervals. Using the maximum (or minimum) within a specified time interval to determine the peak could lead to imprecise peak selection; if, for example, the actual peak is slightly outside these time windows. However, marking each peak manually in large EEG studies (∼25,000 in this study) is not feasible. In order to assess the number of peaks that may be imprecise or unidentifiable, we performed a manual review of the N170 peaks marked at P7 electrode in the face-gaze task (inverted face and averted gaze condition) and in the EoE task (neutral face condition). Out all of the peak selections, these imprecise or unidentifiable peaks account for only 15 (∼2.7%) peaks for the face-gaze (3 in ADHD-only group, 1 in co-occurring autism+ADHD group, 11 in CG) and 26 (∼4.7%) peaks for the EoE task (5 in ADHD-only group, 2 in autism-only group, 1 in co-occurring autism+ADHD group, 18 in CG). Despite the number of imprecise or unidentifiable peaks for the CG being higher, when considering the total number of participants in each group, the percentages in different groups were similar.

### 2.6. Statistical analysis

Outliers were excluded based on the 2*IQR (interquartile range) criterion. Missing ERP variables for each subject (4% for EoE task, and 3.4% for face-gaze task) were imputed separately for each task with the Multivariate Imputation by Chained Equations (mice) R package (57). The variables included as predictors in the multiple imputation specification were sex, age, ADHD diagnosis, autism diagnosis, diagnostic group (based on ADHD and autism diagnosis), emotion, hemisphere, N170 amplitude and latency, P1 amplitude and latency, and P3 amplitude and latency; however, only the ERP measures were specified as variables to be imputed. Fifty multiply imputed datasets were created and each of the 50 datasets were analyzed separately. Then, the results were pooled using the pool function in mice package, which uses the Rubin’s rules (57). Mixed effects models with random intercepts, to control for twin relatedness, were used in the analysis (58). For the EoE task, separate linear mixed models were fitted for N170 amplitude, N170 latency, P1 amplitude or P1 latency as dependent variable, autism (autism+, autism-) and ADHD (ADHD+, ADHD-) as between-subject factors, emotion (anger, disgust, fear, joy, neutral) and hemisphere (right, left) as within-subject factors, and age and sex as covariates. For the face-gaze task, separate linear mixed models were fitted for N170 amplitude, N170 latency, P1 amplitude or P1 latency as dependent variable, autism (autism+, autism-) and ADHD (ADHD+, ADHD-) as between-subject factors, orientation (upright, inverted), direction (direct, averted) and hemisphere (right, left) as within-subject factors, and age and sex as covariates. For both tasks, separate linear mixed models were also fitted for P3 amplitude or P3 latency as dependent variable, autism and ADHD as between-subject factors, emotion or orientation/direction as within-subject factor, and age and sex as covariates. Note that there is no hemisphere within-subject factor for P3 component because it is measured only at midline (Pz) electrode. Whenever age and sex were not significant covariates, the model was fitted again excluding these variables. Note that autism- participants include both CG and ADHD-only groups; autism+ participants include both autism-only and co-occurring autism+ADHD groups; ADHD+ participants include both ADHD-only and co-occurring autism+ADHD groups; ADHD- participants include both CG and autism-only groups. This approach provides a more rigorous way to examine conditions by allowing testing the main effects of autism (regardless of ADHD), main effects of ADHD (regardless of autism), and interactions between autism and ADHD, and has previously been used in other studies using the same tasks in children (23, 31).

Cognitive ability was not included in the models because previous studies indicate that lower average cognitive ability is integral to the ASD phenotype and correcting for this could lead to artefactual positive or negative results (59–62).

Since it is not yet possible to run linear mixed model *post*-*hoc* tests with pooled datasets, we re-ran the linear mixed models with *post hoc* pairwise comparisons (using Bonferroni’s adjustment) on a single imputed dataset, selected always as the first of the 50 imputed datasets. Main and interaction effects consistently showed the same pattern across pooled and single imputed datasets, as well as across imputed and complete case datasets, so here we only report the results from the single imputed dataset. Effect sizes as indexed by Cohen’s d (d_*z*_) were calculated using the R package emmeans for the *post-hoc t*-tests and we report these values throughout the section “3. Results”. Cohen’s d values indicate the following effect sizes: *d*_*z*_ = 0.20, small effect, *d*_*z*_ = 0.50, medium effect, *d*_*z*_ = 0.80, large effect (63).

## 3. Results

Effects involving autism and ADHD are reported below. Effects not related to autism and ADHD, and those related to hemisphere lateralization, are in line with previous literature and are included in the Supplementary material (Supplementary Figures 2–6). For *post-hoc* tests we only report within-group effects and between-group effects within the same condition (emotion, hemisphere, orientation or direction). Moreover, in the EoE task, only arousal-related effects (anger, disgust, fear or joy versus neutral) and valence-related effects (anger, disgust or fear versus joy) are reported. All significant findings are summarized in Table 2.

**TABLE 2:** Summary of the findings for the face-gaze and EoE tasks for different ERP components.

ERP component	Task	Main effects of autism and ADHD	Main effects of task conditions*	Interaction of autism, ADHD, and task conditions	Hemisphere effects and its interactions with autism and ADHD*
P1 amplitude	EoE	–	–	–	left < right
	Face-gaze	–	upright < inverted	–	● left < right ● autism-only: left < right
P1 latency	EoE	autism- < autism+	● joy < anger, disgust, fear ● joy < neutral	● autism-: anger, joy < neutral ● autism+: joy < neutral ● autism- < autism+: anger, disgust, fear ● ADHD+: joy < disgust, fear, neutral	● right < left ● autism- < autism+: left
	Face-gaze	–	–	autism-: upright < inverted	–
N170 amplitude	EoE	autism+ < autism-	● neutral < disgust, fear ● joy < disgust, fear	–	● left < right ● CG, ADHD-only, autism+ADHD: left < right
	Face-gaze	autism+ < autism-	upright < inverted	autism+ < autism-: inverted	left < right
N170 latency	EoE	CG < autism-only	● neutral < anger, disgust, fear ● joy < anger, disgust	● CG: neutral, joy < disgust ● ADHD-only: joy < disgust ● autism+ADHD: neutral, joy < anger, disgust ● autism+ADHD < autism-only: neutral	● right < left ● autism+ADHD: right < left ● autism+ADHD < CG, autism-only: right ● ADHD-only < autism-only: left
	Face-gaze	● autism- < autism+ ● ADHD- < ADHD+	upright < inverted	● CG, autism-only, ADHD-only: upright < inverted ● CG < autism+ADHD: upright ● CG < autism+ADHD: direct ● CG: direct < averted	● CG, ADHD-only < autism+ADHD: direct gaze and left
P3 amplitude	EoE	autism+ < autism-	–	–	–
	Face-gaze	–	upright < inverted	–	–
P3 latency	EoE	–	–	–	–
	Face-gaze	–	–	● autism-: inverted < upright ● autism+ < autism-: upright	–

*Details for main effects of task conditions and hemispheric findings are in Supplementary material.

### 3.1. Expression of emotion (EoE) task

#### 3.1.1. P1 amplitude

**FIGURE 1:**
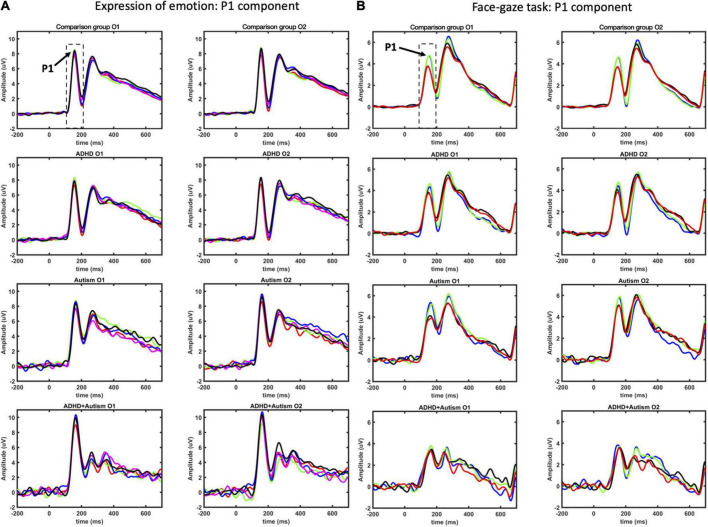
P1 ERP component as measured at O1 and O2 electrodes for EoE **(A)** and face-gaze **(B)** tasks. Time intervals selected for finding the peaks are indicated with dashed lines. For EoE, signals for different emotions are shown with red for anger, green for disgust, blue for fear, magenta for joy, and black for neutral. For face-gaze, signals for different orientations and gaze directions are shown with black for upright face and direct gaze, red for upright face and averted gaze, blue for inverted face and direct gaze, green for inverted face and averted gaze.

**FIGURE 2:**
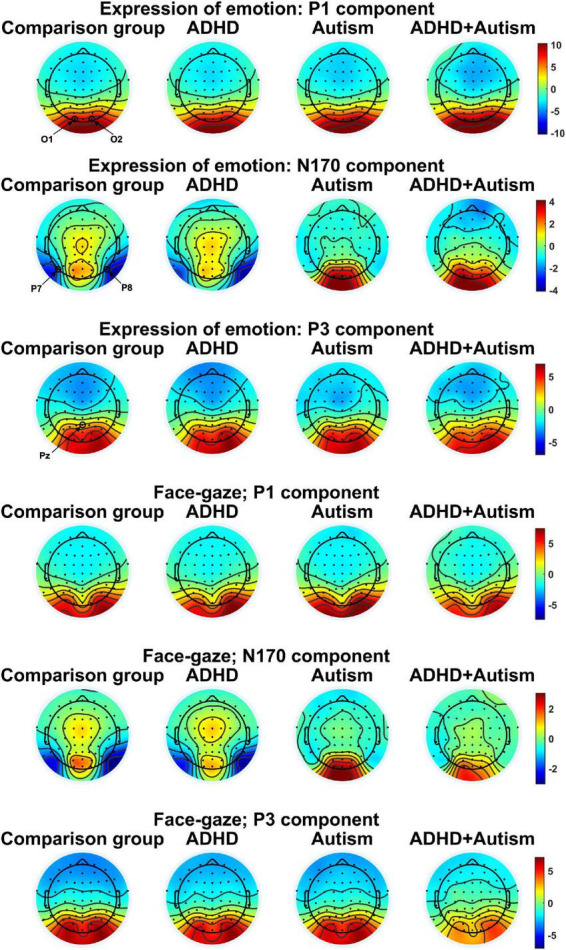
Topographies for the Electroencephalography (EEG) components studied. One topography is plotted per task, EEG variable and group. EEG electrodes O1, O2, P7, P8, and Pz that were used to measure the ERP components are indicated in the figure. Topographies were averaged over different conditions for each task to limit the number of topographies. The topographies were plotted by finding the peak of the grand average signal for each EEG component of interest and then averaging topographies at ± 3.9 ms around the peak.

Event-related potential waveforms for the P1 component are shown in Figure 1A (see Figure 2 for the topographies). Age and sex were not significant covariates and were excluded from the model. No main effects or interactions involving autism or ADHD were significant. Please see Supplementary material for hemisphere related findings.

#### 3.1.2. P1 latency

**FIGURE 3:**
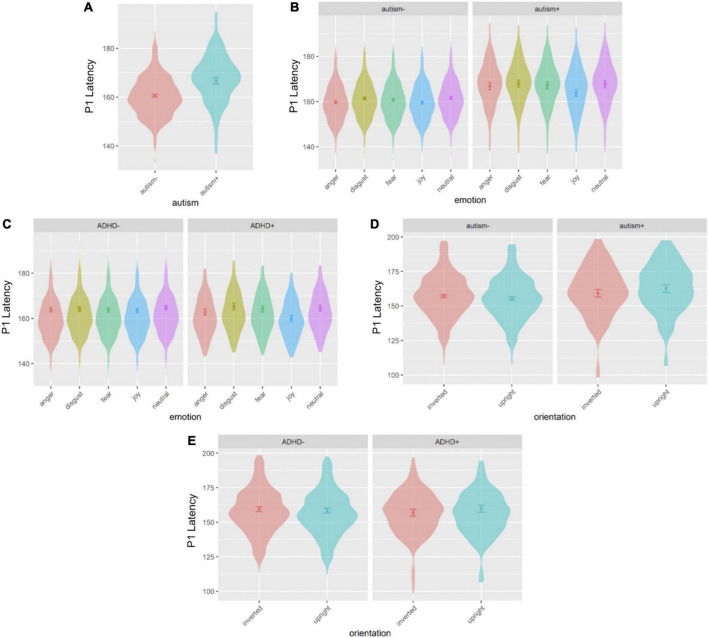
Violin plots of P1 latency showing mean (filled circle), SE (error bars) and frequency of values (width of distribution). EoE task: main effect of autism **(A)**, interaction between autism and emotion **(B)** and interaction between ADHD and emotion **(C)**. Face-gaze task: interaction between autism and orientation **(D)** and interaction between ADHD and orientation **(E)**.

Sex was a significant covariate [*F*(1,539.0) = 4.082, *p* = 0.044], with females showing shorter latencies than males, and was kept in the model while age was not significant and thus excluded. A main effect of autism emerged [*F*(1,530.6) = 14.98, *p* < 0.001], where autism+ individuals showed longer latency than autism- individuals (Figures 1, 3A). There was an interaction effect between autism and emotion [*F*(4,4887.0) = 2.581, *p* = 0.035] (Figure 3B): autism+ individuals showed longer latency specific to negative emotions, the anger, disgust and fear conditions compared to autism- individuals (anger: *p* = 0.002, *d*_*z*_ = 0.898; disgust: *p* = 0.002, *d*_*z*_ = 0.891; fear: *p* = 0.041, *d*_*z*_ = 0.728). Both autism- (*p* = 0.002, *d*_*z*_ = 0.231) and autism+ (*p* = 0.007, *d*_*z*_ = 0.581) individuals showed shorter latency in the joy condition compared to neutral condition. Autism- individuals also showed a shorter latency specific to the anger condition compared to neutral condition (*p* = 0.009, *d*_*z*_ = 0.213). Similarly, there was an interaction effect between ADHD and emotion [*F*(4,4887.0) = 2.478, *p* = 0.042] (Figure 3C), where ADHD+ individuals showed longer latency for disgust and fear compared to joy (disgust-joy: *p* < 0.001, *d*_*z*_ = 0.614; fear-joy: *p* = 0.018, *d*_*z*_ = 0.479), as well as longer latency for neutral compared to joy (*p* < 0.001, *d*_*z*_ = 0.603). All other main effects and interactions involving autism or ADHD (not related to hemisphere lateralization) were not significant.

#### 3.1.3. N170 amplitude

**FIGURE 4:**
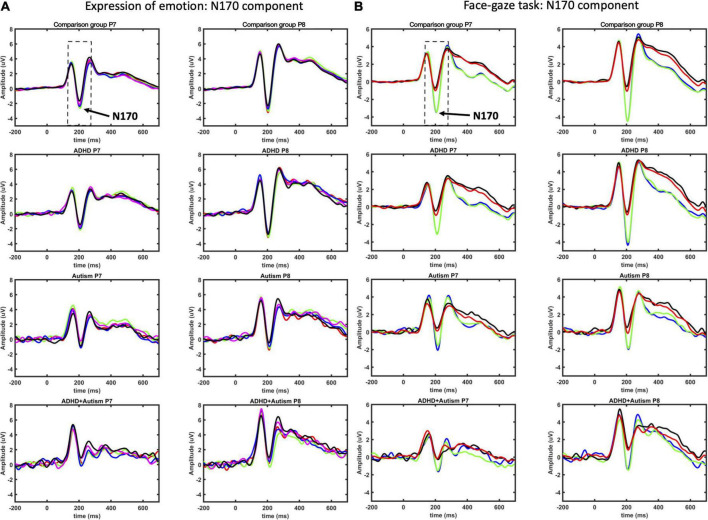
N170 ERP component as measured at P7 and P8 electrodes for EoE **(A)** and face-gaze **(B)** tasks. Time intervals selected for finding the peaks are indicated with dashed lines. For EoE, signals for different emotions are shown with red for anger, green for disgust, blue for fear, magenta for joy, and black for neutral. For face-gaze, signals for different orientations and gaze directions are shown with black for upright face and direct gaze, red for upright face and averted gaze, blue for inverted face and direct gaze, green for inverted face and averted gaze.

**FIGURE 5:**
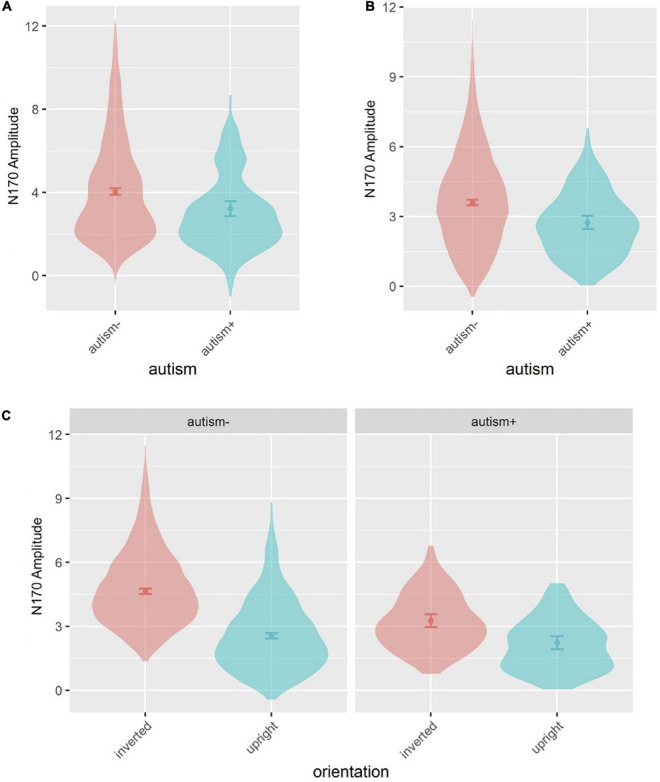
Violin plots of N170 amplitude showing mean (filled circle), SE (error bars) and frequency of values (width of distribution). EoE task: main effect of autism **(A)**. Face-gaze task: main effect of autism **(B)** and interaction between autism and orientation **(C)**.

Age and sex were not significant covariates and were excluded from the model. There was a main effect of autism [*F*(1,492.8) = 5.335, *p* = 0.021], where autism- individuals showed overall greater amplitude than autism+ individuals across all emotions (Figures 4A, 5A). No main effects for ADHD or interactions involving autism or ADHD (and not related to hemisphere lateralization) were significant.

#### 3.1.4. N170 latency

**FIGURE 6:**
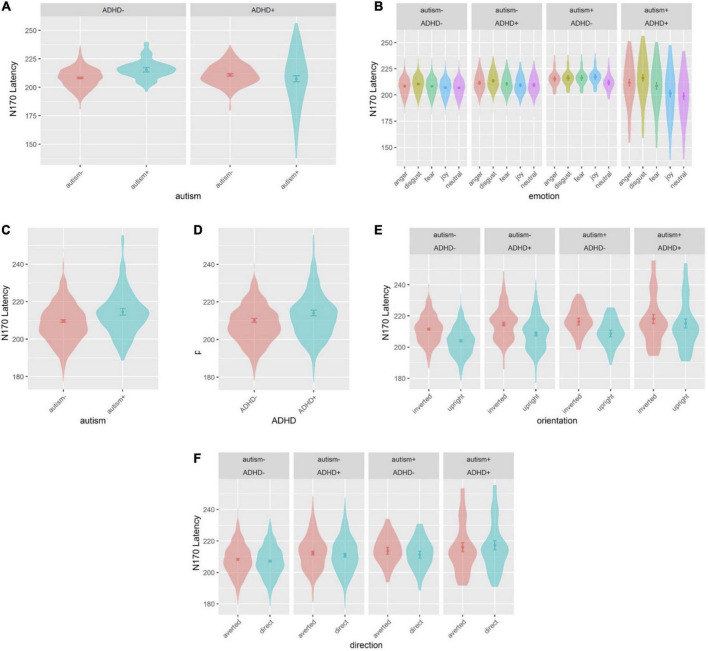
Violin plots of N170 latency showing mean (filled circle), SE (error bars) and frequency of values (width of distribution). EoE task: interaction between autism and ADHD **(A)**, and interaction between autism, ADHD and emotion **(B)**. Face-gaze task: main effect of autism **(C)**, main effect of ADHD **(D)**, interaction between autism, ADHD and orientation **(E)** and interaction between autism, ADHD and direction **(F)**.

Sex was a significant covariate [*F*(1,538.9) = 4.265, *p* = 0.039], with females showing shorter latencies than males, and was kept in the model while age was not significant and was thus excluded. There was a group interaction [*F*(1,537.6) = 9.912, *p* = 0.002] (Figure 6A), where individuals with neither autism nor ADHD (comparison group) showed a shorter latency compared to autism-only individuals (*p* = 0.007, *d*_*z*_ = 0.591). Another interaction emerged between autism and emotion [*F*(4,4887.0) = 2.914, *p* = 0.020], and ADHD and emotion [*F*(4, 4887.0) = 8.089, *p* < 0.001], which were further moderated by an interaction between autism, ADHD and emotion [*F*(4, 4887.0) = 7.185, *p* < 0.001] (Figure 6B): comparison group individuals showed a longer latency for disgust compared to neutral and joy (all *p* < 0.001, *d*_*z*_ disgust-neutral = 0.293, *d*_*z*_ disgust-joy = 0.285); ADHD-only individuals showed longer latency for disgust compared to joy (*p* = 0.038, *d*_*z*_ = 0.385); autism+ADHD individuals showed longer latency for anger and disgust compared to neutral (all *p* < 0.001, *d*_*z*_ anger-neutral = 1.211, *d*_*z*_ disgust-neutral = 1.479), as well as anger and disgust compared to joy (anger-joy: *p* = 0.043, *d*_*z*_ = 0.922; disgust-joy: *p* < 0.001, *d*_*z*_ = 1.189); autism-only individuals showed longer latency in the neutral condition compared to autism+ADHD (*p* = 0.002, *d*_*z*_ = 1.553). All other main effects and interactions involving autism or ADHD were not significant.

#### 3.1.5. P3 amplitude

**FIGURE 7:**
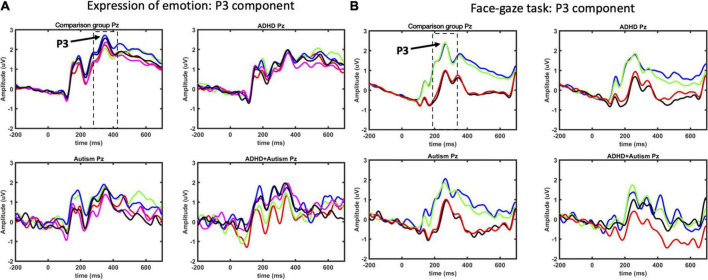
P3 ERP component as measured at Pz electrode for EoE **(A)** and face-gaze **(B)** tasks. Time intervals selected for finding the peaks are indicated with dashed lines. For EoE, signals for different emotions are shown with red for anger, green for disgust, blue for fear, magenta for joy, and black for neutral. For face-gaze, signals for different orientations and gaze directions are shown with black for upright face and direct gaze, red for upright face and averted gaze, blue for inverted face and direct gaze, green for inverted face and averted gaze.

**FIGURE 8:**
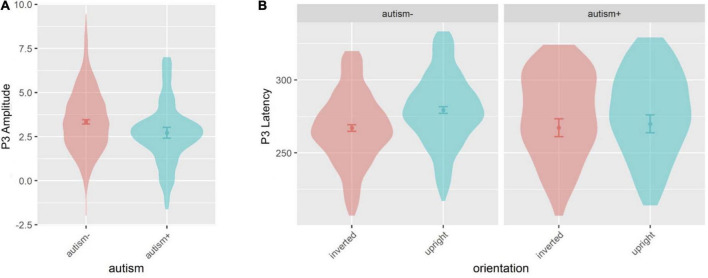
Violin plots of P3 amplitude and P3 latency showing mean (filled circle), SE (error bars), and frequency of values (width of distribution). EoE task: main effect of autism on P3 amplitude **(A)**. Face-gaze task: interaction between autism and orientation on P3 latency **(B)**.

Age and sex were not significant covariates and were excluded from the model. There was a main effect of autism [*F*(1,533.6) = 4.297, *p* = 0.039] (Figures 7A, 8A), where autism- individuals showed greater amplitude than autism+ individuals. All other main effects and interactions involving autism or ADHD were not significant.

#### 3.1.6. P3 latency

For the EoE task, age and sex were not significant covariates and were excluded from the model. All main effects and interactions involving autism or ADHD were not significant.

### 3.2. Face-gaze task

#### 3.2.1. P1 amplitude

Event-related potential waveforms for the P1 component are shown in Figure 1B (see Figure 2 for the topographies). Age and sex were not significant covariates and were excluded from the model. No main effects or interactions involving autism or ADHD were significant. Please see Supplementary Material for hemisphere related findings.

#### 3.2.2. P1 latency

Age and sex were not significant covariates and were excluded from the model. There was an interaction between autism and orientation [*F*(1,3801.0) = 3.956, *p* = 0.047] (Figure 3D), where autism- individuals showed longer latency for inverted faces than for upright faces (*p* = 0.041, *d*_*z*_ = 0.110) but autism+ individuals did not show the same pattern. An interaction emerged between ADHD and orientation [*F*(1,3891.0) = 4.838, *p* = 0.028] (Figure 3E), however, no *post-hoc* tests were significant. This interaction between ADHD and orientation was moderated by an interaction between autism, ADHD and orientation [*F*(1,3801.0) = 6.132, *p* = 0.013], however no *post-hoc* tests were significant. All other main effects and interactions involving autism or ADHD (and not related to hemisphere lateralization) were not significant.

#### 3.2.3. N170 amplitude

Age and sex were not significant covariates and were excluded from the model. There was a main effect of autism [*F*(1,492.4) = 7.973, *p* = 0.005], where autism- individuals showed greater amplitude than autism+ individuals (Figures 4B, 5B). This effect was further moderated by an interaction between autism and orientation [*F*(1,3801.0) = 22.364, *p* < 0.001] (Figure 5C), where both autism+ and autism- individuals showed greater amplitude for inverted faces compared to upright faces (all *p* < 0.001, *d*_*z*_ autism+ = 0.448, *d*_*z*_ autism- = 0.998), but autism- individuals showed greater amplitude for inverted faces compared to autism+ individuals (*p* < 0.001, *d*_*z*_ = 0.665). All other main effects and interactions involving autism or ADHD were not significant.

#### 3.2.4. N170 latency

Sex was a significant covariate [*F*(1,535.3) = 12.65, *p* < 0.001], with females showing shorter latencies than males, and was kept in the model while age was not significant and was excluded from the model. There was a main effect of autism [*F*(1,517.7) = 8.905, *p* = 0.003], where autism- individuals showed shorter latency compared to autism+ individuals (Figure 6C), and a main effect of ADHD [*F*(1,530.2) = 5.677, *p* = 0.017], where ADHD- individuals showed shorter latency than ADHD+ individuals (Figure 6D). There was also an interaction between ADHD and orientation [*F*(1,3801.0) = 5.417, *p* = 0.019], which was further moderated by an interaction between autism, ADHD and orientation [*F*(1, 3801.0) = 5.269, *p* = 0.022] (Figure 6E): the comparison group, autism-only and ADHD-only individuals showed longer latency for inverted faces compared to upright faces (all *p* < 0.001, *d*_*z*_ CG = 0.637, *d*_*z*_ autism-only = 0.778, *d*_*z*_ ADHD-only = 0.633), whereas autism+ADHD individuals showed longer latency for upright faces compared to CG individuals (*p* = 0.002, *d*_*z*_ = 1.065). Finally, there was an interaction between autism, ADHD and direction [*F*(1, 3801.0) = 4.078, *p* = 0.043] (Figure 6F): CG individuals showed a shorter latency than autism+ADHD individuals for direct gaze (*p* = 0.003, *d*_*z*_ = 1.026), and CG individuals showed longer latency for averted than direct gaze (*p* = 0.017, *d*_*z*_ = 0.012). All other main effects and interactions involving autism or ADHD (and not related to hemisphere lateralization) were not significant.

#### 3.2.5. P3 amplitude

Age and sex were not significant covariates and were excluded from the model. All main effects and interactions involving autism or ADHD were not significant.

#### 3.2.6. P3 latency

Sex was a significant covariate [*F*(1,528.0) = 9.997, *p* = 0.002], with females showing shorter latencies than males, and was kept in the model while age was not significant and was excluded from the model. There was an interaction between autism and orientation [*F*(1,1629.0) = 10.19, *p* = 0.001] (Figures 7B, 8B), where autism- individuals showed shorter latency for inverted faces than for upright faces (*p* < 0.001, *d*_*z*_ = 0.381), and autism- individuals showed longer latency for upright faces compared to autism+ individuals (*p* = 0.022, *d*_*z*_ = 0.531). All other main effects and interactions involving autism or ADHD were not significant.

## 4. Discussion

We investigated visual face processing in two passive viewing tasks tapping into different aspects of face, gaze and emotion processing in young adults with autism, ADHD, co-occurring autism and ADHD as well as in a comparison group without ADHD or autism. In line with previous studies using these tasks, we found main effects of emotion on P1 latency, N170 amplitude and N170 latency (Table 2). We further found significantly higher amplitudes for inverted in comparison to upright faces for all three ERP components (P1, N170 and P3) and a longer latency for inverted faces than upright faces for the N170 (Table 2).

The most consistent differences between groups across both tasks were found for the N170 component, where autistic participants showed longer latencies and smaller amplitudes in both tasks in comparison to those without autism. These findings indicate alterations in this neural correlate of face processing for autistic young adults (23, 64) consistent with other studies on autistic adults (25), and some studies in autistic children (22, 23). Furthermore, the finding of a delayed N170 is consistent with studies across both children and adults with autism (25). Similarly, for the face gaze task, young adults with ADHD had a delayed N170, indicating a possible shared atypicality across ADHD and autism in young adulthood. The contrast with previous childhood studies of ADHD could be related to developmental effects on the N170: adults in general have a faster mean latency than children (65). While the mean latency also decreases with age in individuals with autism (and likely ADHD) (25), the lag in individuals with these conditions may become more pronounced over development. In autism, it has been proposed that this may indicate a compounding of social atypicalities so that the childhood symptoms lead to reduced engagement with social stimuli and contribute to a failure to develop neural specialization in social processing by adulthood (66).

Early studies of the N170 failed to show any effect of emotional expressions on its amplitude or latency [e.g., (67)], which was taken as evidence that this component is a correlate of mechanisms specifically involved in the structural encoding of faces (68). More recent evidence indicates, however, that the N170 amplitude is sensitive to emotional expressions (21). While in this study, we did not find differential modulation of N170 amplitude by emotions, we did find modulation of latency with delays for emotional face stimuli compared to neutral, and further that negative emotions (i.e., anger and disgust) elicited longer delays compared to positive face stimuli (i.e., joy). The relevance of emotional expressions to features of the N170, as well as modulation by other contextual affective information, such as body expressions and prosody of speech (69, 70), has led to interpretations of the N170 indexing the interplay between several sources of information at the more basic structural level and higher level information, including facial expression (71).

Specific task differences for the N170 emerged for individuals with co-occurring autism and ADHD, who showed longer N170 latencies compared to comparison group individuals for the direct gaze and not for the averted gaze condition indicating lack of modulation by gaze direction. Similarly, we found that N170 latencies were longer in the co-occurring group compared to the comparison group for upright faces. The existence of this effect only for the upright face condition and not for the inverted faces might indicate that the co-occurring group is less affected by the more challenging inverted face condition. This lack of inversion effect has previously been reported in children with autism (72). A childhood study showed a reduced inversion effect for those with co-occurring ADHD and autism compared to typically developing and autism only for P1 latency but not for N170 latency (31). Overall, our findings indicate that those with co-occurring autism and ADHD show the same pattern of atypical processing as those with single diagnoses with additional differences indexed by reduced sensitivity to face inversion and gaze. The fact that these effects were only observed for the co-occurring group might indicate additional or amplified atypicalities in comparison to ADHD and autism alone (23). This is in agreement with less favorable cognitive, emotional and functional outcomes reported co-occurring autism and ADHD in adulthood (4–6).

In line with previous studies of adults, autistic participants showed a delayed P1 latency compared to those without autism (here, specific to the EoE task) (24). The P1 latency of young adults with autism was also shown to be longer in anger, disgust and fear conditions in comparison to those without autism. Significant differences in P1 latency are more limited in studies on autistic children (22, 23, 73) and in Batty et al. the difference in P1 latency between autistic and CG children was not retained after matching for verbal equivalent age (74). The only childhood study reporting P1 latency differences in autism was Tye et al. with shorter P1 latency to direct gaze in comparison to averted gaze for the autistic group (31). The longer P1 latency observed here only for the EoE task suggests that this difference might not be due to a general slowing of stimuli processing in autism (75). The significant interactions between diagnostic group and emotion for EoE task, and between diagnostic group and orientation for face-gaze task provides evidence that the P1 may be differentially modulated even at this early stage of face processing. The modulation of P1 amplitude with emotion has previously been reported (76) but the current finding of modulation of P1 latency by emotion is novel.

In our study we observed shorter latencies in females in comparison to males for N170 (in both tasks), P1 (EoE task) and P3 (face-gaze task) components. The findings for N170 and P100 latencies are in line with previous studies, and might support the theory that women are faster in detecting face stimuli and structural face encoding during face processing in comparison to men (77, 78).

Contrary to expectations, young adults with ADHD did not show a reduction in P3 amplitude; however, those with autism showed reduced P3 amplitude compared to those without autism in the EoE task across all emotions. Other P3 effects specific to autism indicated longer latencies for upright faces compared to those without autism. These findings are in contrast to previous studies in both ADHD and autism but there is some evidence from other modalities that facial processing atypicalities exist in autism beyond early processing stages (79, 80). The P3 is considered as an index of more top-down processing including emotional evaluation and memory encoding (28, 32). Reduced P3 amplitudes in autistic adults may reflect diminished facilitation of evaluation of emotional stimuli in the autism sample or an atypicality in resource capacity for processing these stimuli. The slower P3 latency only for upright faces and not for inverted faces in autism could arguably be due to less reliance on global information and more on local/detailed information (74, 81).

This study has one of the largest EEG datasets of young adults with ADHD and autism; however, possibly due to use of strict diagnostic criteria (ADOS-2 and DIVA-2), the number of co-occurring ADHD and autism cases (16) is limited which could affect the power to detect effects specific to the co-occurring group. We aimed to represent those who would fulfill diagnosis in a clinical setting in this study, but future studies could include individuals with subthreshold symptoms of autism and ADHD, which is likely to increase detection of individuals with symptoms of both conditions. The prevalence of these conditions in adulthood has been suggested to be an underestimate as, while the diagnostic criteria are reduced in adulthood, clinical observations indicate that the symptoms manifest differently into adulthood or the symptoms can be masked by adaptive or compensatory skills (82, 83). As detailed in section “2. Materials and methods”, automatic detection of ERP component peaks as it was done in this study might lead to marking some imprecise peaks and the precision of some of these peaks could be improved by using more advanced algorithms (84). Importantly, however, a manual review indicated that the percentage of imprecise or unidentifiable peaks was very small (4.7 and 2.7% of the total number of peaks) and these percentages do not differ much between groups. Furthermore, any outliers would have been removed prior to the statistical analysis.

## 5. Conclusion

This study provides a comprehensive examination of the temporal stages of face processing using salient socio-emotional stimuli: emotional expression, face gaze and orientation. We found consistent differences between those with autism and without in the N170 component across stimuli, with the autistic group showing lower amplitude and a delayed peak for N170, in agreement with some previous studies in adulthood. Furthermore, autistic participants had longer P1 latencies and smaller P3 amplitudes in response to emotional expressions and longer P3 latencies for upright faces compared to those without autism. Alterations for the ADHD group were limited to longer N170 latencies, specific to the face-gaze task, overlapping with the same finding in autism. Those with co-occurring autism and ADHD shared these atypicalities with the autism and ADHD participants but had additional differences in gaze modulation and a lack of an inversion effect as indexed by the N170. These findings suggest that there are identifiable and measurable socio-functional atypicalities in young adults with autism and ADHD that could be used along with other measures understand the progression of these conditions. Future work should investigate how much these neurophysiological atypicalities contribute to functional and social outcomes to clarify if they could serve as potential treatment targets in broader remediation interventions (85).

## Data availability statement

The raw data supporting the conclusions of this article will be made available by the authors, without undue reservation.

## Ethics statement

The studies involving human participants were reviewed and approved by the King’s College London Psychiatry, Nursing and Midwifery Research Ethics Subcommittee. The patients/participants provided their written informed consent to participate in this study. Written informed consent was obtained from the individual(s) for the publication of any identifiable images or data included in this article.

## Author contributions

ÜA, RC, and GM conceived and designed the analysis. ÜA and RC performed the analysis and wrote the initial draft in consultation with GM and CT. GM designed the study and acquired funding. All authors provided feedback on the manuscript, read, and approved the final version of this manuscript.

## Footnotes

1
www.nsgportal.org

## References

[1] Arnett J. Emerging Adulthood: what is it, and what is it good for? *Child Dev Perspect.* (2007) 1:68–73. 10.1111/j.1750-8606.2007.00016.x

[2] Stroud C Walker L Davis M Irwin C. Investing in the health and well-being of young adults. *J Adolesc Health.* (2015) 56:127–9. 10.1016/j.jadohealth.2014.11.012 25620297

[3] Lau-Zhu A Fritz A McLoughlin G. Overlaps and distinctions between attention deficit/hyperactivity disorder and autism spectrum disorder in young adulthood: systematic review and guiding framework for EEG-imaging research. *Neurosci Biobehav Rev.* (2019) 96:93–115. 10.1016/j.neubiorev.2018.10.009 30367918 PMC6331660

[4] Anckarsäter H Stahlberg O Larson T Hakansson C Jutblad S Niklasson L The impact of adhd and autism spectrum disorders on temperament, character, and personality development. *AJP.* (2006) 163:1239–44. 10.1176/ajp.2006.163.7.1239 16816230

[5] Murray M. Attention-deficit/hyperactivity disorder in the context of autism spectrum disorders. *Curr Psychiatry Rep.* (2010) 12:382–8. 10.1007/s11920-010-0145-3 20694583

[6] van der Meer J Oerlemans A van Steijn D Lappenschaar M de Sonneville L Buitelaar J Are autism spectrum disorder and attention-deficit/hyperactivity disorder different manifestations of one overarching disorder? cognitive and symptom evidence from a clinical and population-based sample. *J Am Acad Child Adolesc Psychiatry.* (2012) 51:1160–72.e3. 10.1016/j.jaac.2012.08.024 23101742

[7] Bora E Pantelis C. Meta-analysis of social cognition in attention-deficit/hyperactivity disorder (ADHD): comparison with healthy controls and autistic spectrum disorder. *Psychol Med.* (2016) 46:699–716. 10.1017/S0033291715002573 26707895

[8] Ros R Graziano P. Social functioning in children with or at risk for attention deficit/hyperactivity disorder: a meta-analytic review. *J Clin Child Adolesc Psychol.* (2018) 47:213–35. 10.1080/15374416.2016.1266644 28128989

[9] Aydin Ü Capp S Tye C Colvert E Lau-Zhu A Rijsdijk F Quality of life, functional impairment and continuous performance task event-related potentials (ERPs) in young adults with ADHD and autism: a twin study. *JCPP Adv.* (2022) 2:e12090. 10.1002/jcv2.12090 PMC10242939 37431386

[10] Greene R Biederman J Faraone S Sienna M Garcia-Jetton J. Adolescent outcome of boys with attention-deficit/hyperactivity disorder and social disability: results from a 4-year longitudinal follow-up study. *J Consult Clin Psychol.* (1997) 65:758–67. 10.1037/0022-006X.65.5.758 9337495

[11] Helles A Gillberg C Gillberg C Billstedt E. Asperger syndrome in males over two decades: stability and predictors of diagnosis. *J Child Psychol Psychiatry.* (2015) 56:711–8. 10.1111/jcpp.12334 25283685

[12] Neary P Gilmore L Ashburner J. Post-school needs of young people with high-functioning Autism Spectrum Disorder. *Res Autism Spectr Disord.* (2015) 18:1–11. 10.1016/j.rasd.2015.06.010

[13] Tobin M Drager K Richardson LF. A systematic review of social participation for adults with autism spectrum disorders: support, social functioning, and quality of life. *Res Autism Spectr Disord.* (2014) 8:214–29. 10.1016/j.rasd.2013.12.002

[14] Wilhelm O Herzmann G Kunina-Habenicht O Danthiir V Schacht A Sommer W. Individual differences in perceiving and recognizing faces—One element of social cognition. *J Personal Soc Psychol.* (2010) 99:530–48. 10.1037/a0019972 20677889

[15] Mikami A Miller M Lerner M. Social functioning in youth with attention-deficit/hyperactivity disorder and autism spectrum disorder: transdiagnostic commonalities and differences. *Clin Psychol Rev.* (2019) 68:54–70. 10.1016/j.cpr.2018.12.005 30658861

[16] Dima D Perry G Messaritaki E Zhang J Singh K. Spatiotemporal dynamics in human visual cortex rapidly encode the emotional content of faces. *Hum Brain Mapp.* (2018) 39:3993–4006. 10.1002/hbm.24226 29885055 PMC6175429

[17] Luo W Feng W He W Wang N Luo Y. Three stages of facial expression processing: erp study with rapid serial visual presentation. *Neuroimage.* (2010) 49:1857–67. 10.1016/j.neuroimage.2009.09.018 19770052 PMC3794431

[18] Rossion B. Understanding face perception by means of human electrophysiology. *Trends Cogn Sci.* (2014) 18:310–8. 10.1016/j.tics.2014.02.013 24703600

[19] McPartland J. Considerations in biomarker development for neurodevelopmental disorders. *Curr Opin Neurol.* (2016) 29:118–22. 10.1097/WCO.0000000000000300 26844621 PMC4798424

[20] Bentin S Allison T Puce A Perez E McCarthy G. Electrophysiological studies of face perception in humans. *J Cogn Neurosci.* (1996) 8:551–65. 10.1162/jocn.1996.8.6.551 20740065 PMC2927138

[21] Hinojosa J Mercado F Carretié L. N170 sensitivity to facial expression: a meta-analysis. *Neurosci Biobehav Rev.* (2015) 55:498–509. 10.1016/j.neubiorev.2015.06.002 26067902

[22] Hileman C Henderson H Mundy P Newell L Jaime M. Developmental and individual differences on the P1 and N170 ERP components in children with and without autism. *Dev Neuropsychol.* (2011) 36:214–36. 10.1080/87565641.2010.549870 21347922 PMC3724226

[23] Tye C Battaglia M Bertoletti E Ashwood K Azadi B Asherson P Altered neurophysiological responses to emotional faces discriminate children with ASD, ADHD and ASD+ADHD. *Biol Psychol.* (2014) 103:125–34. 10.1016/j.biopsycho.2014.08.013 25179537

[24] O’Connor K Hamm J Kirk I. The neurophysiological correlates of face processing in adults and children with Asperger’s syndrome. *Brain Cogn.* (2005) 59:82–95. 10.1016/j.bandc.2005.05.004 16009478

[25] Kang E Keifer C Levy E Foss-Feig J McPartland J Lerner M. Atypicality of the N170 Event-related potential in autism spectrum disorder: a meta-analysis. *Biol Psychiatry Cogn Neurosci Neuroimaging.* (2018) 3:657–66. 10.1016/j.bpsc.2017.11.003 30092916 PMC6089230

[26] Key A Corbett B. The unfulfilled promise of the N170 as a social biomarker. *Biol Psychiatry Cogn Neurosci Neuroimaging.* (2020) 5:342–53. 10.1016/j.bpsc.2019.08.011 31679960 PMC7064396

[27] Williams L Hermens D Palmer D Kohn M Clarke S Keage H Misinterpreting emotional expressions in attention-deficit/hyperactivity disorder: evidence for a neural marker and stimulant effects. *Biol Psychiatry.* (2008) 63:917–26. 10.1016/j.biopsych.2007.11.022 18272140

[28] Raz S Dan O. Altered event-related potentials in adults with ADHD during emotional faces processing. *Clin Neurophysiol.* (2015) 126:514–23. 10.1016/j.clinph.2014.06.023 25018010

[29] Ibáñez A Petroni A Urquina H Torrente F Torralva T Hurtado E Cortical deficits of emotional face processing in adults with ADHD: its relation to social cognition and executive function. *Soc Neurosci.* (2011) 6:464–81. 10.1080/17470919.2011.620769 21961874

[30] Rossion B Caharel S. ERP evidence for the speed of face categorization in the human brain: disentangling the contribution of low-level visual cues from face perception. *Vis Res.* (2011) 51:1297–311. 10.1016/j.visres.2011.04.003 21549144

[31] Tye C Mercure E Ashwood K Azadi B Asherson P Johnson M Neurophysiological responses to faces and gaze direction differentiate children with ASD, ADHD and ASD + ADHD. *Dev Cogn Neurosci.* (2013) 5:71–85. 10.1016/j.dcn.2013.01.001 23466656 PMC6987819

[32] Hajcak G MacNamara A Olvet D. Event-related potentials, emotion, and emotion regulation: an integrative review. *Dev Neuropsychol.* (2010) 35:129–55. 10.1080/87565640903526504 20390599

[33] Schindler S Bublatzky F. Attention and emotion: an integrative review of emotional face processing as a function of attention. *Cortex.* (2020) 130:362–86. 10.1016/j.cortex.2020.06.010 32745728

[34] Kaiser A Aggensteiner P Baumeister S Holz N Banaschewski T Brandeis D. Earlier versus later cognitive event-related potentials (ERPs) in attention-deficit/hyperactivity disorder (ADHD): a meta-analysis. *Neurosci Biobehav Rev.* (2020) 112:117–34. 10.1016/j.neubiorev.2020.01.019 31991190

[35] McLoughlin G Gyurkovics M Aydin Ü. What has been learned from using EEG methods in research of ADHD? In: Stanford SC Sciberras E editors. *Current topics in behavioral neurosciences.* Berlin: Springer (2022). p. 1–30. 10.1007/7854_2022_344 35637406

[36] Stavropoulos K Viktorinova M Naples A Foss-Feig J McPartland J. Autistic traits modulate conscious and nonconscious face perception. *Soc Neurosci.* (2018) 13:40–51. 10.1080/17470919.2016.1248788 27750521 PMC6194504

[37] Farroni T Johnson M Csibra G. Mechanisms of eye gaze perception during infancy. *J Cogn Neurosci.* (2004) 16:1320–6. 10.1162/0898929042304787 15509381

[38] Shephard E Milosavljevic B Mason L Elsabbagh M Tye C Gliga T Neural and behavioural indices of face processing in siblings of children with autism spectrum disorder (ASD): a longitudinal study from infancy to mid-childhood. *Cortex.* (2020) 127:162–79. 10.1016/j.cortex.2020.02.008 32200288 PMC7254063

[39] Tye C Bussu G Gliga T Elsabbagh M Pasco G Johnsen K Understanding the nature of face processing in early autism: a prospective study. *J Abnorm Psychol.* (2020) 131:542–55. 10.1037/abn0000648 35901386 PMC9330670

[40] Battaglia M Ogliari A Zanoni A Citterio A Pozzoli U Giorda R Influence of the serotonin transporter promoter gene and shyness on children’s cerebral responses to facial expressions. *Arch Gen Psychiatry.* (2005) 62:85–94. 10.1001/archpsyc.62.1.85 15630076

[41] Battaglia M Ogliari A Zanoni A Villa F Citterio A Binaghi F Children’s discrimination of expressions of emotions: relationship with indices of social anxiety and shyness. *J Am Acad Child Adolesc Psychiatry.* (2004) 43:358–65. 10.1097/00004583-200403000-00019 15076270

[42] Haworth C Davis O Plomin R. Twins Early Development Study (TEDS): a genetically sensitive investigation of cognitive and behavioral development from childhood to young adulthood. *Twin Res Hum Genet.* (2013) 16:117–25. 10.1017/thg.2012.91 23110994 PMC3817931

[43] Rimfeld K Malanchini M Spargo T Spickernell G Selzam S McMillan A Twins early development study: a genetically sensitive investigation into behavioral and cognitive development from infancy to emerging adulthood. *Twin Res Hum Genet.* (2019) 22:508–13. 10.1017/thg.2019.56 31544730 PMC7056571

[44] Capp S Agnew-Blais J Lau-Zhu A Colvert E Tye C Aydin Ü Is quality of life related to high autistic traits, high adhd traits and their interaction? evidence from a young-adult community-based twin sample. *J Autism Dev Disord.* (2022). [Epub ahead of print]. 10.1007/s10803-022-05640-w 35802291 PMC10465683

[45] Colvert E Tick B McEwen F Stewart C Curran S Woodhouse E Heritability of autism spectrum disorder in a UK population-based twin sample. *JAMA Psychiatry.* (2015) 72:415–23. 10.1001/jamapsychiatry.2014.3028 25738232 PMC4724890

[46] Tye C Rijsdijk F Greven C Kuntsi J Asherson P McLoughlin G. Shared genetic influences on ADHD symptoms and very low-frequency EEG activity: a twin study. *J Child Psychol Psychiatry.* (2012) 53:706–15. 10.1111/j.1469-7610.2011.02501.x 22118296 PMC3859923

[47] Kooij JJS. *Adult ADHD: Diagnostic Assessment and Treatment. London: Springer* (2013). 10.1007/978-1-4471-4138-9

[48] American Psychiatric Association [APA]. *Diagnostic and statistical manual of mental disorders (DSM-5§).* Arlington, VA: American Psychiatric Pub (2013). p. 1414.

[49] Hus V Lord C. The autism diagnostic observation schedule, module 4: revised algorithm and standardized severity scores. *J Autism Dev Disord.* (2014) 44:1996–2012. 10.1007/s10803-014-2080-3 24590409 PMC4104252

[50] Tottenham N Tanaka J Leon A McCarry T Nurse M Hare T The NimStim set of facial expressions: judgments from untrained research participants. *Psychiatry Res.* (2009) 168:242–9. 10.1016/j.psychres.2008.05.006 19564050 PMC3474329

[51] Grice SJ Halit H Farroni T Baron-Cohen S Bolton P Johnson MH. Neural correlates of eye-gaze detection in young children with autism. *Cortex* (2005) 41:342–53. 10.1016/S0010-9452(08)70271-5 15871599

[52] Delorme A Makeig S. EEGLAB: an open source toolbox for analysis of single-trial EEG dynamics including independent component analysis. *J Neurosci Methods.* (2004) 134:9–21. 10.1016/j.jneumeth.2003.10.009 15102499

[53] Palmer J Kreutz-Delgado K Makeig S. *AMICA: an adaptive mixture of independent component analyzers with shared components. Tech. Report.* San Diego: Swartz Center for Computational Neuroscience (2011). p. 15.

[54] Martínez-Cancino R Delorme A Truong D Artoni F Kreutz-Delgado K Sivagnanam S The open EEGLAB portal Interface: high-Performance computing with EEGLAB. *NeuroImage.* (2021) 224:116778. 10.1016/j.neuroimage.2020.116778 32289453 PMC8341158

[55] Winkler I Debener S Müller K Tangermann M. On the influence of high-pass filtering on ICA-based artifact reduction in EEG-ERP. *Annu Int Conf IEEE Eng Med Biol Soc.* (2015) 2015:4101–5. 10.1109/EMBC.2015.7319296 26737196

[56] Bigdely-Shamlo N Kreutz-Delgado K Kothe C Makeig S. EyeCatch: data-mining over Half a Million EEG independent components to construct a fully-automated eye-component detector. *Conf Proc IEEE Eng Med Biol Soc.* (2013) 2013:5845–8. 10.1109/EMBC.2013.6610881 24111068 PMC4136453

[57] Van Buuren S Groothuis-Oudshoorn K. mice: multivariate Imputation by Chained Equations in R. *J Stat Softw.* (2011) 45:1–67. 10.18637/jss.v045.i03

[58] Malone S Burwell S Vaidyanathan U Miller M McGue M Iacono W. Heritability and molecular-genetic basis of resting EEG activity: a genome-wide association study. *Psychophysiology.* (2014) 51:1225–45. 10.1111/psyp.12344 25387704 PMC4262140

[59] Dennis M Francis D Cirino P Schachar R Barnes M Fletcher J. Why IQ is not a covariate in cognitive studies of neurodevelopmental disorders. *J Int Neuropsychol Soc.* (2009) 15:331–43. 10.1017/S1355617709090481 19402919 PMC3075072

[60] Lukito S O’Daly O Lythgoe D Whitwell S Debnam A Murphy C Neural correlates of duration discrimination in young adults with autism spectrum disorder, attention-deficit/hyperactivity disorder and their comorbid presentation. *Front Psychiatry.* (2018) 9:569. 10.3389/fpsyt.2018.00569 30487760 PMC6246684

[61] Miller G Chapman J. Misunderstanding analysis of covariance. *J Abnorm Psychol.* (2001) 110:40–8. 10.1037/0021-843X.110.1.40 11261398

[62] Postema M van Rooij D Anagnostou E Arango C Auzias G Behrmann M Altered structural brain asymmetry in autism spectrum disorder in a study of 54 datasets. *Nat Commun.* (2019) 10:4958. 10.1038/s41467-019-13005-8 31673008 PMC6823355

[63] Cohen J. *Statistical power analysis for the behavioral sciences.* 2nd ed. New York, NY: Routledge (1988). p. 567. 10.4324/9780203771587

[64] Itier R Latinus M Taylor M. Face, eye and object early processing: what is the face specificity? *NeuroImage.* (2006) 29:667–76. 10.1016/j.neuroimage.2005.07.041 16169749

[65] Taylor M McCarthy G Saliba E Degiovanni E. ERP evidence of developmental changes in processing of faces. *Clin Neurophysiol.* (1999) 110:910–5. 10.1016/S1388-2457(99)00006-1 10400205

[66] Dawson G. Early behavioral intervention, brain plasticity, and the prevention of autism spectrum disorder. *Dev Psychopathol.* (2008) 20:775–803. 10.1017/S0954579408000370 18606031

[67] Eimer M Holmes A McGlone F. The role of spatial attention in the processing of facial expression: an ERP study of rapid brain responses to six basic emotions. *Cogn Affect Behav Neurosci.* (2003) 3:97–110. 10.3758/CABN.3.2.97 12943325

[68] Eimer M Holmes A. Event-related brain potential correlates of emotional face processing. *Neuropsychologia.* (2007) 45:15–31. 10.1016/j.neuropsychologia.2006.04.022 16797614 PMC2383989

[69] de Gelder B den Stock J. Real faces, real emotions: perceiving facial expressions in naturalistic contexts of voices, bodies, and scenes. In: Calder A Rhodes G Johnson M Haxby J editors. *Oxford handbook of face perception.* New York, NY: Oxford University Press (2011). 10.1093/oxfordhb/9780199559053.013.0027

[70] Wieser M Brosch T. Faces in context: a review and systematization of contextual influences on affective face processing. *Front Psychol.* (2012) 3:471. 10.3389/fpsyg.2012.00471 23130011 PMC3487423

[71] George N. The facial expression of emotions. In: Armony J Vuilleumier P. *The Cambridge handbook of human affective neuroscience.* New York, NY: Cambridge University Press (2013). p. 171–97. 10.1017/CBO9780511843716.011

[72] McPartland J Dawson G Webb S Panagiotides H Carver L. Event-related brain potentials reveal anomalies in temporal processing of faces in autism spectrum disorder. *J Child Psychol Psychiatry.* (2004) 45:1235–45. 10.1111/j.1469-7610.2004.00318.x 15335344

[73] Parker T Crowley M Naples A Rolison M Wu J Trapani J The N170 event-related potential reflects delayed neural response to faces when visual attention is directed to the eyes in youths with ASD. *Autism Res.* (2021) 14:1347–56. 10.1002/aur.2505 33749161

[74] Batty M Meaux E Wittemeyer K Rogé B Taylor M. Early processing of emotional faces in children with autism: an event-related potential study. *J Exp Child Psychol.* (2011) 109:430–44. 10.1016/j.jecp.2011.02.001 21458825

[75] Vettori S Jacques C Boets B Rossion B. Can the N170 Be used as an electrophysiological biomarker indexing face processing difficulties in autism spectrum disorder? *Biol Psychiatry Cogn Neurosci Neuroimaging.* (2019) 4:321–3. 10.1016/j.bpsc.2018.07.015 30391291

[76] Blechert J Sheppes G Di Tella C Williams H Gross J. See what you think: reappraisal modulates behavioral and neural responses to social stimuli. *Psychol Sci.* (2012) 23:346–53. 10.1177/0956797612438559 22431908

[77] Sun T Li L Xu Y Zheng L Zhang W Zhou F Electrophysiological evidence for women superiority on unfamiliar face processing. *Neurosci Res.* (2017) 115:44–53. 10.1016/j.neures.2016.10.002 27794442

[78] Nowparast Rostami H Hildebrandt A Sommer W. Sex-specific relationships between face memory and the N170 component in event-related potentials. *Soc Cogn Affect Neurosci.* (2020) 15:587–97. 10.1093/scan/nsaa059 32367139 PMC7328020

[79] Davies M Dapretto M Sigman M Sepeta L Bookheimer S. Neural bases of gaze and emotion processing in children with autism spectrum disorders. *Brain Behav.* (2011) 1:1–11. 10.1002/brb3.6 22398976 PMC3217668

[80] Philip R Whalley H Stanfield A Sprengelmeyer R Santos I Young A Deficits in facial, body movement and vocal emotional processing in autism spectrum disorders. *Psychol Med.* (2010) 40:1919–29. 10.1017/S0033291709992364 20102666

[81] Deruelle C Rondan C Gepner B Tardif C. Spatial frequency and face processing in children with autism and asperger syndrome. *J Autism Dev Disord.* (2004) 34:199–210. 10.1023/B:JADD.0000022610.09668.4c 15162938

[82] Adler L Cohen J. Diagnosis and evaluation of adults with attention-deficit/hyperactivity disorder. *Psychiatr Clin.* (2004) 27:187–201. 10.1016/j.psc.2003.12.003 15063992

[83] Lai M Baron-Cohen S. Identifying the lost generation of adults with autism spectrum conditions. *Lancet Psychiatry.* (2015) 2:1013–27. 10.1016/S2215-0366(15)00277-1 26544750

[84] Lopez-Calderon J Luck S. ERPLAB: an open-source toolbox for the analysis of event-related potentials. *Front Hum Neurosci.* (2014) 8:213. 10.3389/fnhum.2014.00213 24782741 PMC3995046

[85] Eack S Hogarty S Greenwald D Litschge M Porton S Mazefsky C Cognitive enhancement therapy for adult autism spectrum disorder: results of an 18-month randomized clinical trial. *Autism Res.* (2018) 11:519–30. 10.1002/aur.1913 29286586 PMC5867220

