# Supplementary material for: Face processing in young adults with autism and ADHD: An event related potentials study
Source: Front Psychiatry. 2023 Mar 14;14:1080681. doi: 10.3389/fpsyt.2023.1080681 (PMC10043418; doi:10.3389/fpsyt.2023.1080681)
Supplement: Supplementary file 1 [file Data_Sheet_1.pdf]

Supplementary Material

Supplementary methods

The medical exclusions were same as those used in the Twins Early Development Study (TEDS) (1). These exclusions were conditions in the following categories: 1) severe ASD (Cases that are non-verbal or with severely delayed speech, or with difficulties in completing activities.); 2) severe cerebral palsy; 3) chromosomal disorders; 4) inherited or single-gene disorders associated with mental impairment; 5) brain damage or disorders affecting brain function; 6) profound deafness or complete blindness; 7) global developmental delay. More details on these exclusions could be found in related TEDS webpage (2). In addition to these, all participants recruited had an estimated IQ of 70 or above (measured during adolescence). This was to ensure that all participants could perform all the tasks and questionnaires successfully. Furthermore all participants have normal or corrected to normal vision to enable them participating in the study.

Supplementary results

Effects not involving autism and ADHD are reported below. Effects related to autism and ADHD are included in the main text, except for those related to hemisphere lateralisation, which are also reported below. Note in the EoE task, only arousal-related effects (anger, disgust, fear or joy versus neutral) and valence-related effects (anger, disgust or fear versus joy) are reported.

Expression of Emotion (EoE) Task

P1 Amplitude

Age and sex were not significant covariates and were excluded from the model. There was a main effect of hemisphere ($F(1,4887.0) = 28.14$, $p < .001$) (Figure S2A), with greater amplitude for the right hemisphere than the left hemisphere. There was a significant interaction between autism and hemisphere ($F(1, 4887.0) = 4.229$, $p = .039$) (Figure S2B), where both autism- and autism+ individuals showed greater amplitude in the right hemisphere than left hemisphere (all $p < .001$, d_z autism- = .169, d_z autism+ = .382). All other main effects and interactions involving autism or ADHD were not significant.

P1 Latency

Sex was a significant covariate ($F(1,539.0) = 4.082$, $p = .044$), with females showing shorter latencies than males, and was kept in the model while age was not significant and was excluded from the model. There was a main effect of emotion ($F(1,4887.0) = 7.723$, p <

.001) (Figure S3A): anger, disgust and fear showed longer latency than joy (anger- joy: p = .026, d_z = .247; disgust- joy: p < .001, d_z = .381; fear- joy: p = .005, d_z = .286), and joy showed shorter latency than neutral (p < .001, d_z = .406). There was also a main effect of hemisphere (F(1,4887.0) = 33.08, p < .001) (Figure S3B), with longer latency for the left hemisphere compared to the right hemisphere. There was an interaction between autism and hemisphere (F(1,4887.0) = 7.118, p = .008) (Figure S3C), where autism+ individuals and autism- individuals showed longer latency for the left hemisphere compared to right hemisphere (all p < .001, d_z autism- = .160, d_z autism+ = .437), and autism- individuals showed shorter latency in the left hemisphere compared to autism+ individuals (p < .001, d_z = .892). All other main effects and interactions not involving autism or ADHD were not significant.

N170 Amplitude

Age and sex were not significant covariates and were excluded from the model. There was a main effect of emotion (F(1,4887.0) = 5.889, p < .001) (Figure S4A): disgust and fear showed greater amplitude than neutral (disgust-neutral: p = .019, d_z = .256; fear-neutral: p = .027, d_z = .246) and joy (disgust-joy: p = .002, d_z = .309; fear-joy: p = .002, d_z = .299). There was also a main effect of hemisphere (F(1,4887.0) = 37.06, p < .001) (Figure S4B), with greater amplitude for the right hemisphere compared to the left hemisphere. There was an interaction between ADHD and hemisphere (F(1,4887.0) = 20.912, p < .001), which was further moderated by an interaction between autism, ADHD and hemisphere (F(1, 4887.0) = 6.982, p = .008) (Figure S4C): CG individuals, ADHD-only individuals and autism+ADHD individuals showed greater amplitude in the right hemisphere compared to left hemisphere (all p < .001, d_z CG = .239, d_z ADHD-only = .440, d_z autism+ADHD = .667). All other main effects and interactions not involving autism or ADHD were not significant.

N170 Latency

Sex was a significant covariate (F(1,538.9) = 4.265, p = .039) , with females showing shorter latencies than males, and was kept in the model while age was not significant and was excluded from the model. There was a main effect of emotion (F(1,4887.0) = 14.66, p < .001) (Figure S5A): anger, disgust and fear showed longer latency than neutral (anger-neutral: p < .001, dz = .375; disgust-neutral: p < .001, dz = .555; fear-neutral: p = .002, dz = .303), and anger and disgust showed longer latency than joy (anger-joy: p = .006, dz = .284; disgust-joy: p < .001, dz = .464). There was also a main effect of hemisphere (F(1,4887.0) = 107.5, p < .001) (Figure S5B), with longer latency for the left hemisphere compared to the right hemisphere. There was an interaction between autism and hemisphere (F(1, 4887.0) = 68.29, p < .001), and ADHD and hemisphere (F(1, 4887.0) = 77.50, p < .001), which were

further moderated by an interaction between autism, ADHD and Hemisphere ($F(1, 4887.0) = 49.84$, $p < .001$) (Figure S5C): autism+ADHD individuals showed longer latency in the left hemisphere compared to right hemisphere ($p < .001$, $d_z = 1.792$); CG and autism-only individuals showed longer latency in the right hemisphere compared to autism+ADHD (all $p < .001$, d_z CG - autism+ADHD = 1.064, d_z autism-only - autism+ADHD = 1.592); autism-only individuals showed longer latency in the left hemisphere compared to CG individuals ($p = .017$, $d_z = .653$). All other main effects and interactions not involving autism or ADHD were not significant.

P3 Amplitude

Age and sex were not significant covariates and were excluded from the model. All main effects and interactions not involving autism or ADHD were not significant.

P3 Latency

Age and sex were not significant covariates and were excluded from the model. All main effects and interactions not involving autism or ADHD were not significant.

Face-gaze Task

P1 Amplitude

Age and sex were not significant covariates and were excluded from the model. There was a main effect of orientation ($F(1,3801.0) = 23.27$, $p < .001$) (Figure S2C), with greater amplitude for inverted faces compared to upright faces. There was also a main effect of hemisphere ($F(1,3801.0) = 6.767$, $p = .009$) (Figure S2D), with greater amplitude for the right hemisphere compared to left hemisphere. There was an interaction effect between ADHD and hemisphere ($F(1, 3801.0) = 6.159$, $p = .013$), which was further moderated by an interaction between autism, ADHD and hemisphere ($F(1, 3801.0) = 7.144$, $p = .007$) (Figure S3): autism-only individuals showed attenuated amplitude in the left hemisphere compared to the right hemisphere (p = .003, $d_z = .496$). All other main effects and interactions involving autism or ADHD were not significant.

P1 Latency

Age and sex were not significant covariates and were excluded from the model. There was an interaction between autism, ADHD, orientation and hemisphere (F(1,3801.0) = 3.927, p = .048), however no post-hoc tests were significant for either interaction. All other main effects and interactions not involving autism or ADHD were not significant.

N170 Amplitude

Age and sex were not significant covariates and were excluded from the model. There was a main effect of orientation (F(1,3801.0) = 154.8, p < .001) (Figure S4D), with greater amplitude for inverted faces compared to upright faces. There was also a main effect of hemisphere (F(1,3801.0) = 22.21, p < .001) (Figure S4E), with greater amplitude for the right hemisphere than the left hemisphere. All other main effects and interactions not involving autism or ADHD were not significant.

N170 Latency

Sex was a significant covariate (F(1,535.3) = 12.65, p < .001), with females showing shorter latencies than males, and was kept in the model while age was not significant and was excluded from the model. There was a main effect of orientation (F(1,3801.0) = 97.09, p < .001) (Figure S5D), with longer latency for inverted faces than upright faces. There was also an interaction between autism and hemisphere (F(1,535.3) = 5.745, p = .016), which was further moderated by an interaction between autism, ADHD, direction and hemisphere (F(1, 3801.0) = 5.781, p = .016) (Figure S5E): autism+ADHD individuals showed longer latency for direct gaze in the left hemisphere compared to CG individuals (p < .001, d_z = 1.429) and ADHD-only individuals (p = .025, d_z = 1.149). All other main effects and interactions not involving autism or ADHD were not significant.

P3 Amplitude

For the face-gaze task, age and sex were not significant covariates and were excluded from the model. There was a main effect of orientation (F(1,1629.0) = 219.4, p < .001) (Figure S6), with greater amplitude for inverted faces compared to upright faces. All other main effects and interactions not involving autism or ADHD were not significant.

P3 Latency

Sex was a significant covariate (F(1,528.0) = 9.997, p = .002), with females showing shorter latencies than males, and was kept in the model while age was not significant and was

excluded from the model. All main effects and interactions not involving autism or ADHD were not significant.

References

1. Haworth CMA, Davis OSP, Plomin R. Twins Early Development Study (TEDS): a genetically sensitive investigation of cognitive and behavioral development from childhood to young adulthood. *Twin Res Hum Genet* (2013) 16:117–125. doi: 10.1017/thg.2012.91

2. TEDS Exclusions. https://www.teds.ac.uk/datadictionary/exclusions.htm [Accessed February 7, 2022]

3. Tottenham N, Tanaka JW, Leon AC, McCarry T, Nurse M, Hare TA, Marcus DJ, Westerlund A, Casey B, Nelson C. The NimStim set of facial expressions: Judgments from untrained research participants. *Psychiatry Research* (2009) 168:242–249. doi: 10.1016/j.psychres.2008.05.006

4. Farroni T, Johnson MH, Csibra G. Mechanisms of Eye Gaze Perception during Infancy. *Journal of Cognitive Neuroscience* (2004) 16:1320–1326. doi: 10.1162/0898929042304787

5. Grice SJ, Halit H, Farroni T, Baron-Cohen S, Bolton P, Johnson MH. Neural Correlates of Eye-Gaze Detection in Young Children with Autism. *Cortex* (2005) 41:342–353. doi: 10.1016/S0010-9452(08)70271-5

Table S1: Mean (Standard deviation) of number epochs retained for each group for each condition.

Condition	CG	ADHD	Autism	Autism+ADHD
**EoE; Anger**	38.0 (3.1)	37.0 (3.9)	36.7 (3.1)	38.5 (1.8)
**EoE; Disgust**	38.1 (2.9)	37.0 (4.0)	37.2 (2.9)	38.4 (1.9)
**EoE; Fear**	38.0 (2.9)	37.2 (3.8)	36.6 (3.0)	37.8 (3.1)
**EoE; Joy**	38.2 (2.8)	37.0 (4.1)	36.8 (2.8)	38.1 (3.1)
**EoE; Neutral**	37.7 (3.2)	37.0 (3.8)	36.7 (3.6)	37.0 (3.4)
**Face-gaze; Inverted face Direct gaze**	56.4 (4.6)	54.0 (7.6)	54.7 (7.4)	54.6 (5.5)
**Face-gaze; Inverted face Averted gaze**	112.3 (9.7)	108.1 (13.3)	109.7 (13.8)	110.5 (10.3)
**Face-gaze; Upright face Direct gaze**	56.4 (4.6)	53.9 (7.2)	54.3 (7.1)	55.6 (5.0)
**Face-gaze; Upright face Averted gaze**	112.6 (8.7)	108.0 (13.8)	108.5 (14.2)	111.5 (9.8)

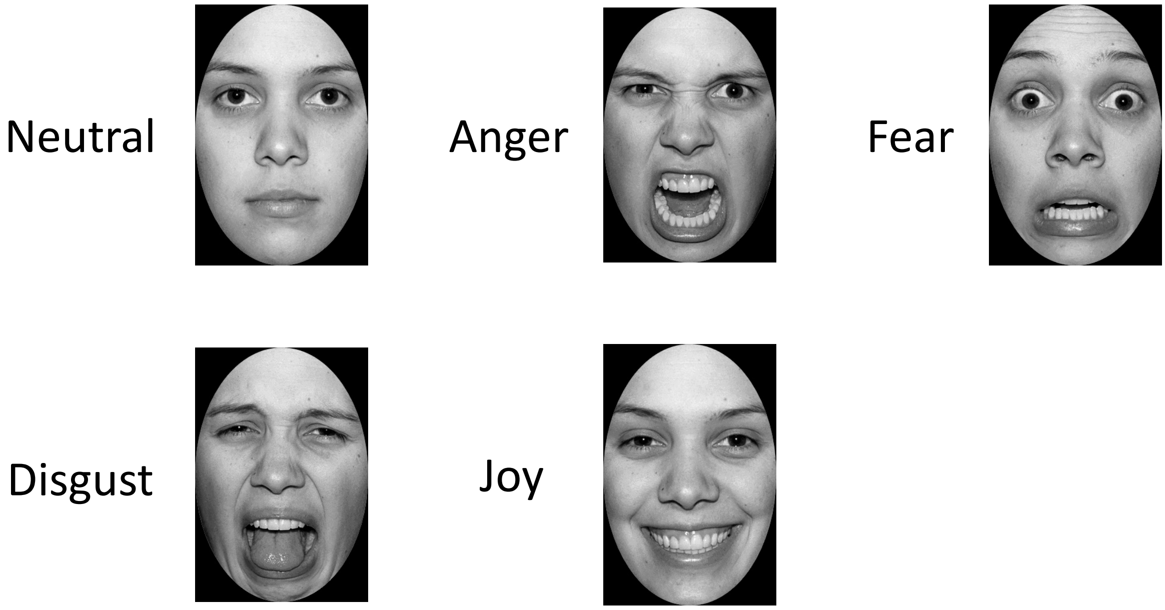

***Figure S1**: Examples of face images shown in expression of emotion task. Pictures of stimuli with female presented here; the task included both male and female stimuli. The images were modified from the NimStim set of facial expressions (3). The images used for the face-gaze task could be found in Grice et al. (Figure 1) and Farroni et al. (Figure 3, colour version is used in this study) (4,5).*

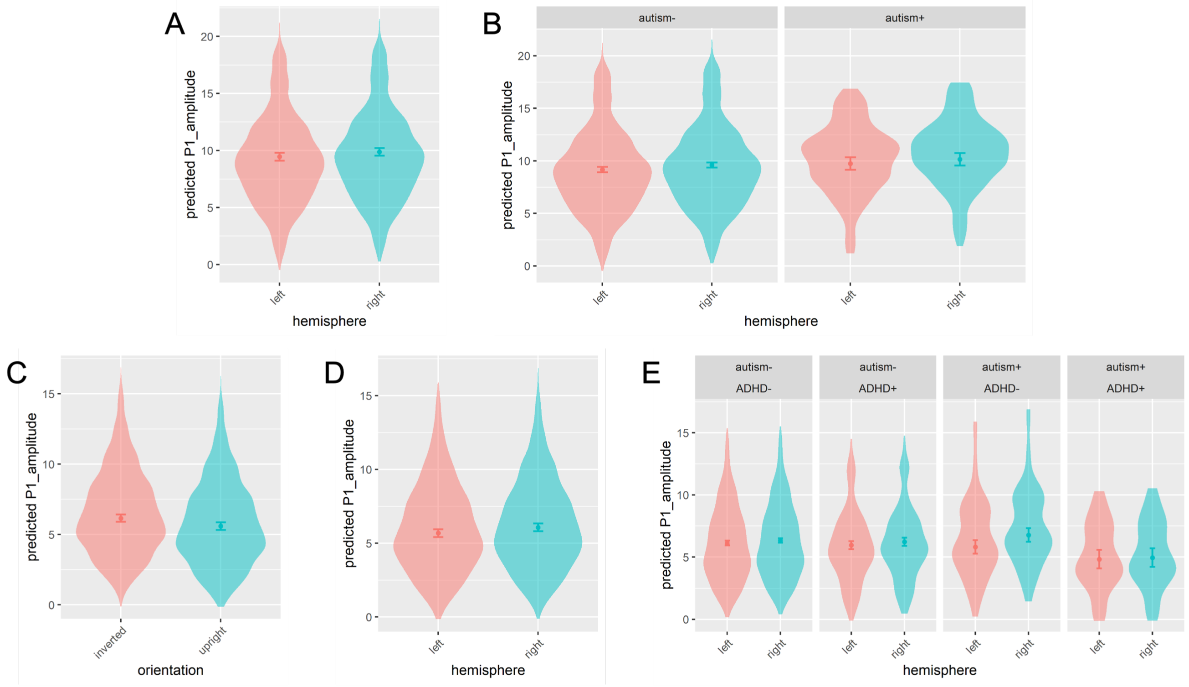

Figure S2. Violin plots of P1 amplitude showing mean (filled circle), SE (error bars) and frequency of values (width of distribution). EoE task: main effect of hemisphere (A) and interaction between autism and hemisphere (B). Face-gaze task: main effect of orientation (C), hemisphere (D) and interaction between autism, ADHD and hemisphere (E).

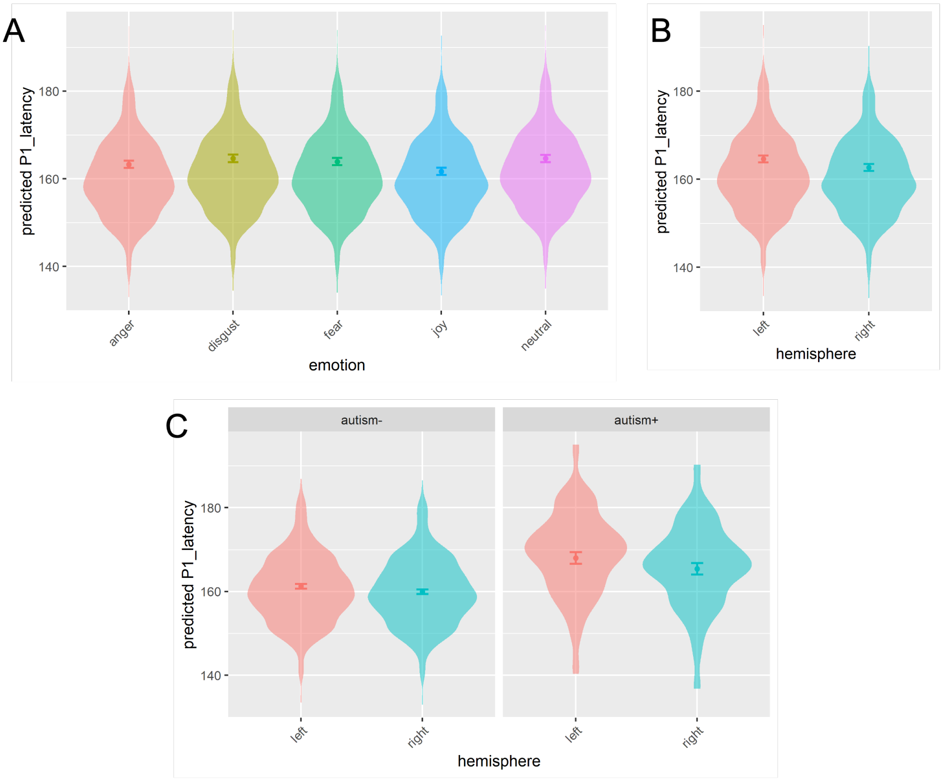

Figure S3. *Violin plots of P1 latency showing mean (filled circle), SE (error bars) and frequency of values (width of distribution). EoE task: main effect of emotion (A) and hemisphere (B), and interaction between autism and hemisphere (C).*

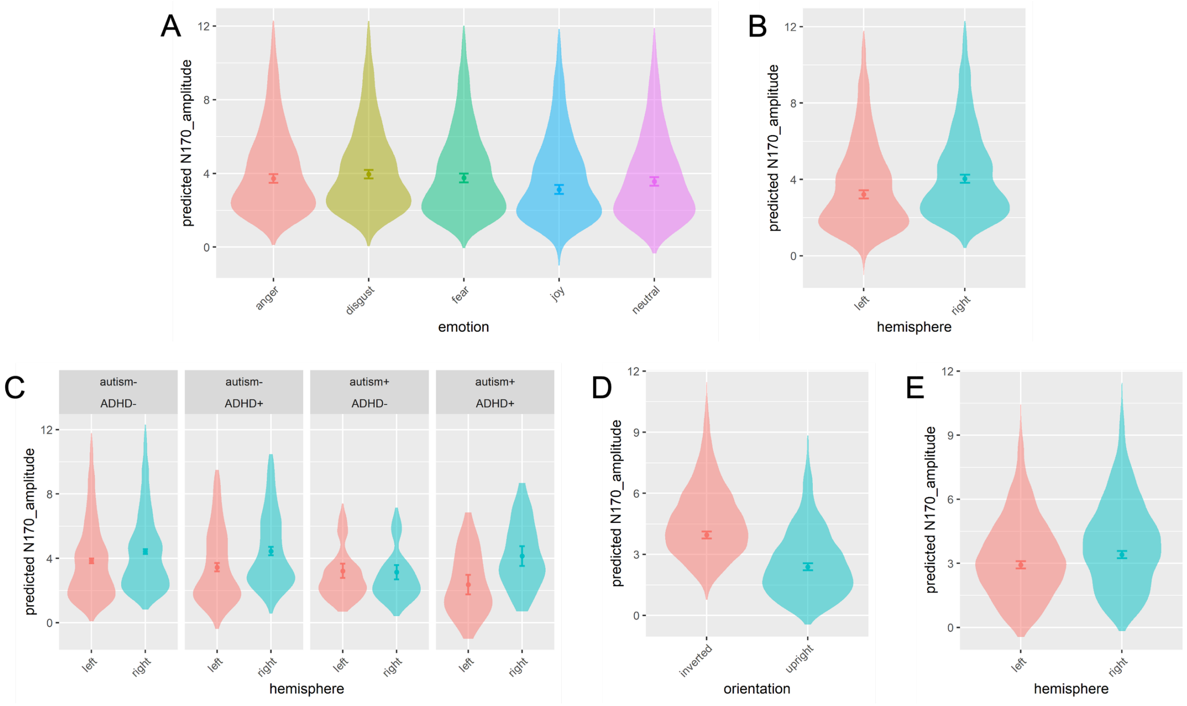

Figure S4. *Violin plots of N170 amplitude showing mean (filled circle), SE (error bars) and frequency of values (width of distribution). EoE task: main effect of emotion (A) and hemisphere (B), and interaction between autism, ADHD and hemisphere (C). Face-gaze task: main effect of orientation (D) and hemisphere (E).*

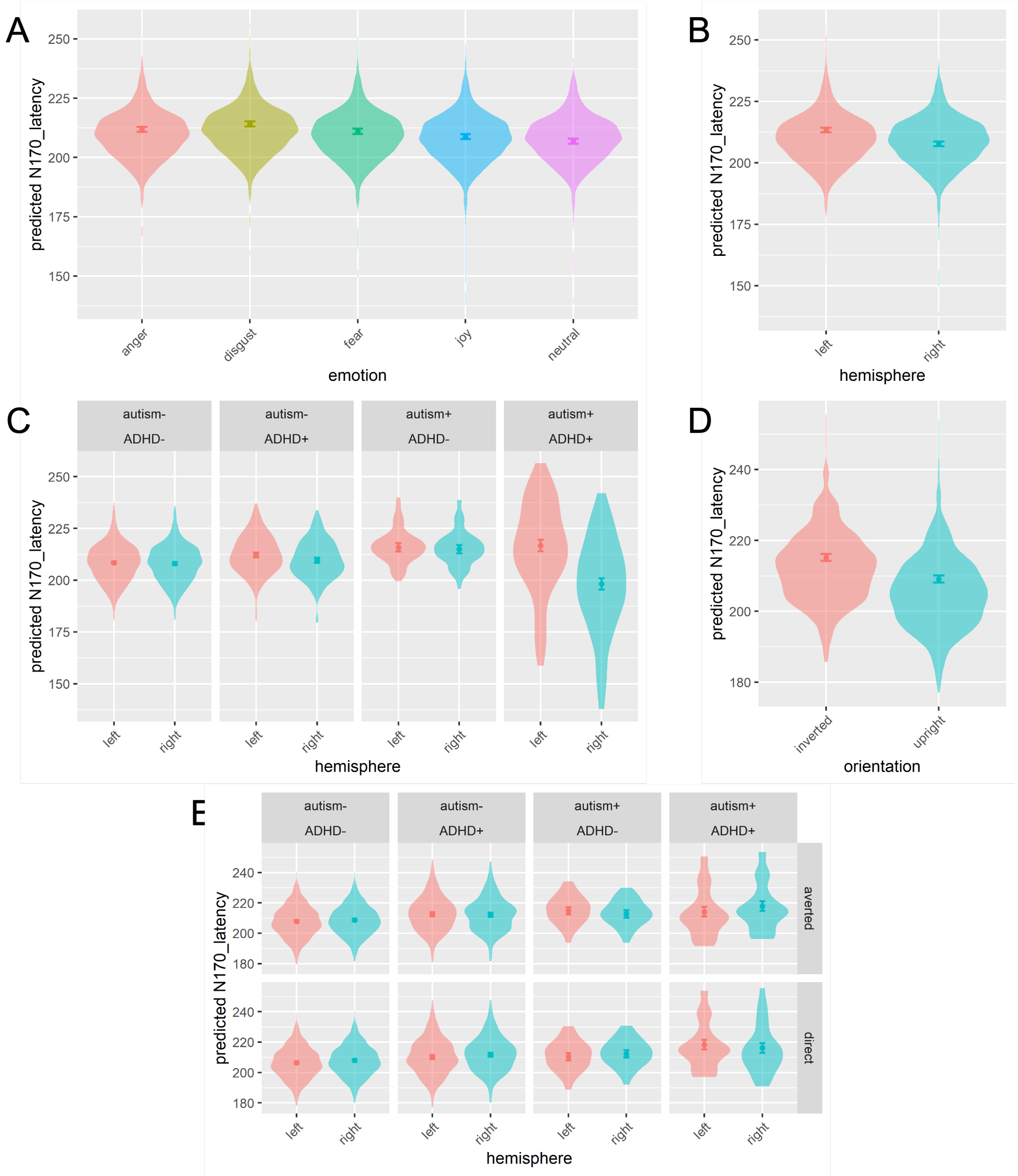

Figure S5. *Violin plots of N170 latency showing mean (filled circle), SE (error bars) and frequency of values (width of distribution). EoE task: main effect of emotion (A) and hemisphere (B), and interaction between autism, ADHD and hemisphere (C). Face-gaze task: main effect of orientation (D) and interaction between autism, ADHD, direction and hemisphere (E).*

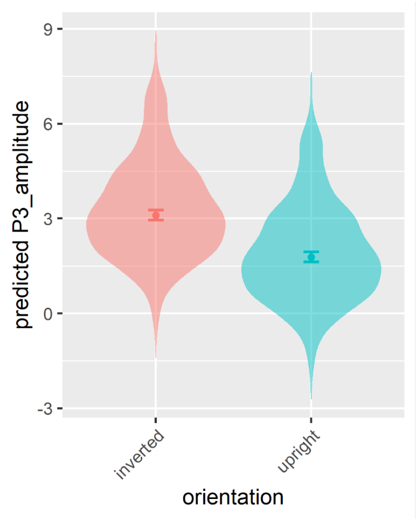

Figure S6. *Violin plots of P3 amplitude showing mean (filled circle), SE (error bars) and frequency of values (width of distribution). Face-gaze task: main effect of orientation.*
